# A novel nomogram to predict 90-day mortality in patients with hepatitis B virus-related acute-on-chronic liver failure: a single-center retrospective study

**DOI:** 10.1186/s12876-023-02727-1

**Published:** 2023-03-25

**Authors:** Ye Xiong, Zuoxun Xia, Lu Yang, Jianrong Huang

**Affiliations:** 1 The Department of Infectious Diseases, State Key Laboratory for Diagnosis and Treatment of Infectious Diseases, National Clinical Research Center for Infectious Diseases, Collaborative Innovation Center for Diagnosis and Treatment of Infectious Diseases, The First Affiliated Hospital, Zhejiang University School of Medicine, 79 Qingchun Road, Hangzhou, 310003 China; 2 Guizhou Medical University, Guiyang, Guizhou, China

**Keywords:** Acute-on-chronic hepatitis B liver failure, Nomogram, Prognosis, Mortality

## Abstract

**Background:**

Acute-on-chronic liver failure (ACLF) is a critical illness with high mortality. Herein, we developed and validated a new and simple prognostic nomogram to predict 90-day mortality in hepatitis B virus-related ACLF (HBV-ACLF) patients.

**Methods:**

This single-center retrospective study collected data from 181 HBV-ACLF patients treated between June 2018 and March 2020. The correlation between clinical data and 90-day mortality in patients with HBV-ACLF was assessed using univariate and multivariate logistic regression analyses.

**Results:**

Multivariate logistic regression analysis showed that age (*p* = 0.011), hepatic encephalopathy (*p* = 0.001), total bilirubin (*p* = 0.007), international normalized ratio (*p* = 0.006), and high-density lipoprotein cholesterol (*p* = 0.011) were independent predictors of 90-day mortality in HBV-ACLF patients. A nomogram was created to predict 90-day mortality using these risk factors. The C-index for the prognostic nomogram was calculated as 0.866, and confirmed to be 0.854 via bootstrapping verification. The area under the curve was 0.870 in the external validation cohort. The predictive value of the nomogram was similar to that of the Chinese Group on the Study of Severe Hepatitis B score, and exceeded the performance of other prognostic scores.

**Conclusion:**

The prognostic nomogram constructed using the factors identified in multivariate regression analysis might serve as a beneficial tool to predict 90-day mortality in HBV-ACLF patients.

## Introduction

Acute-on-chronic liver failure (ACLF) is a multifaceted condition characterized by progressive deterioration of liver function, multiple organ failure, poor treatment effects, and high short-term mortality [1]. The short-(1–3 months) and medium-term (6 months) mortality of patients with ACLF can reach as high as 50–90%, and the 28-day mortality is 15 fold higher than that of patients with chronic liver disease (CLD) alone [2]. Despite the present popularity of vaccines, hepatitis B virus (HBV) infection still inflicts a devastating impact on human health, affecting approximately 240 million people worldwide [3, 4]. HBV-related ACLF (HBV-ACLF) is a serious complication of chronic hepatitis B (CHB) infection, and can develop at any stage of disease progression [5]. In China, HBV is the major cause of CLD. Moreover, it is estimated that HBV-ACLF accounts for more than 70% of ACLF cases in the region, causing over 100,000 deaths every year [6, 7].

Currently, the treatment of ACLF mainly involves the use of an artificial liver support system (ALSS), cell therapies, and liver transplant (LT) [8]. Nevertheless, an accumulating body of research indicates that ALSS use exerts no significant benefit on the survival of patients with end-stage liver failure [9, 10]. Moreover, because of ethical and safety concerns, the clinical application of cell therapies has so far been limited [8, 11]. In many countries, LT has been applied as a medical intervention to save the lives of patients with ACLF. However, because of the shortage of donor liver grafts, identifying suitable recipients and prioritizing LT is crucial [12].

To date, several predictive scoring systems have been developed to evaluate the prognosis of patients with ACLF, including the Model for End-stage Liver Disease (MELD) score, Chronic Liver Failure Consortium Organ Failure score (CLIF-C OFs), CLIF-C ACLFs, and Chinese Group on the Study of Severe Hepatitis B score (COSSH-ACLFs). However, the MELD score, CLIF-C Ofs, and CLIF-C ACLFs were all established in Western countries, in which the populations are over 70% Caucasians. Furthermore, the main causes of disease were hepatitis C virus and alcohol, rather than HBV. Hence, it is still challenging to use these scoring systems to predict HBV-ACLF prognosis [13, 14]. On the other hand, although the COSSH-ACLFs is a predictive scoring model based on HBV infection, its ability to accurately predict the prognosis of patients with HBV-ACLF is uncertain and requires further verification [15]. In addition, the COSSH-ACLFs also involves multiple parameters and requires complex organ failure assessments. Thus, a more simple prognostic model, which would be more feasible in clinical practice, is needed.

The purpose of this study was to establish a valid but simple prediction tool using laboratory and clinical parameters to predict 90-day mortality in patients with HBV-ACLF and provide guidance for clinical treatment and decision-making.

## Materials and methods

### Patients

**Fig. 1:**
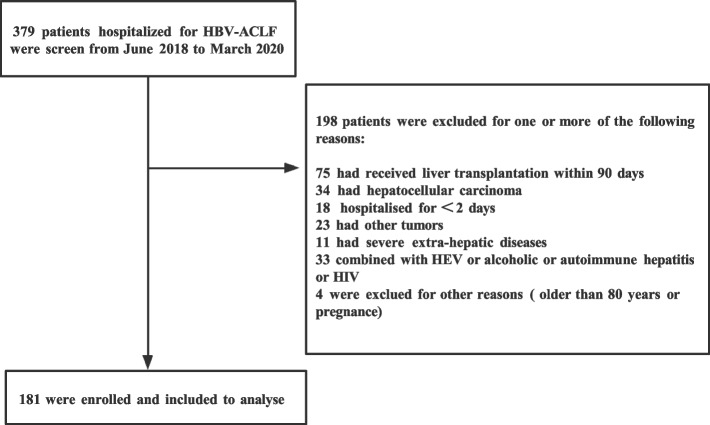
Flowchart of the screening and enrollment of patients with HBV-ACLF. Abbreviations: HBV-ACLF, hepatitis B virus-related acute-on-chronic liver failure; HEV, hepatitis E virus; HIV, human immunodeficiency virus

For this single-center, retrospective study, we extracted data from all patients diagnosed with HBV-ACLF during admission in the First Affiliated Hospital of Zhejiang University School of Medicine from June 2018 to March 2020. HBV-ACLF was diagnosed based on the COSSH-ACLF criteria [15]. The inclusion criteria were as follows: (1) age between 18 and 80 years; (2) hospitalization for at least 2 days; (3) ACLF caused by HBV infection; (4) serum total bilirubin (TBIL) ≥ 12 mg/dL; and (5) international normalized ratio (INR) ≥ 1.5. The exclusion criteria were as follows: (1) ACLF resulting from other causes of liver damage, such as hepatitis A, C, D, and E viruses, alcohol, or autoimmune hepatitis; (2) liver cancer or other tumors; (3) coinfection with human immunodeficiency virus; (4) pregnant women; (5) severe cardiopulmonary diseases or previous renal failure; (6) incomplete data or loss to follow-up; and (7) patients who underwent LT. Based on these eligibility criteria, 181 patients were enrolled in this study (Fig. 1).

All patients included in this study received comprehensive treatments, including antiviral therapy for HBV-DNA-positive patients, as well as treatment for hypoproteinemia, infection, hepatic encephalopathy (HE), ascites, electrolyte disorder, and other complications.

### Data collection

We extracted patient data before treatment from the medical records, including clinical (age, sex, body mass index [BMI], oxygen saturation, and blood pressure) and laboratory (white blood cell [WBC], hemoglobin, platelet count, albumin, alanine aminotransferase, aspartate aminotransferase, TBIL level, creatinine [Cr], serum sodium, INR, D-dimer, total cholesterol [TC], triglyceride, high-density lipoprotein cholesterol [HDL-C], low-density lipoprotein cholesterol, alpha fetoprotein [AFP], and HBV DNA) data, as well as data on the presence of ongoing complications such as HE, upper gastrointestinal bleeding (UGB), infection, and ascites. Furthermore, all enrolled patients were followed up from the date of their enrollment until the end of the 90-day follow-up, or death.

Several ACLF predictive scores were calculated using the following formula: MELD = 9.57 $\times$ Ln (Cr, mg/dL) + 3.78 $\times$ Ln (TBIL, mg/dL) + 11.2 $\times$ Ln (INR) + 6.43 [16]; CLIF-C-OFs including TBIL, Cr, the grade of HE, INR, mean arterial pressure, and respiratory status were evaluated according to the standard criteria: CLIF-C-ACLF = 10 $\times$ [0.33 $\times$ CLIF-OFs + 0.04 $\times$ Age + 0.63 $\times$ Ln(WBC)-2] [14]; COSSH-ACLFs = 0.741 $\times$ INR + 0.523 $\times$ HBV-SOFA + 0.026 $\times$ Age + 0.003 $\times$ TBIL [15].

### Statistical analysis

SPSS software (version 26.0) was used for all statistical analyses. Continuous variables are presented as the means ± standard deviation or medians with interquartile range, and were analyzed using the independent two-sample *t*-test and Mann–Whitney *U* test, respectively. Categorical variables are presented as frequencies, and were analyzed using Pearson’s chi-squared test. Univariate and multivariate logistic regression models were also used to assess the correlation between clinical variables and 90-day mortality in patients with HBV-ACLF to screen for independent predictors, which were subsequently introduced into R v.3.6.1 (http://www.r-project.org/) to establish a nomogram prediction model. A receiver-operating characteristic curve (ROC) was drawn, and the area under the curve (AUC) was calculated to evaluate the discriminatory ability of the nomogram prediction model. Decision curve analysis was then conducted to determine the clinical applicability of the nomogram [17]. The bootstrap repeated sampling method (1000 bootstrap resampling) was used for internal verification. The corrected C-index was calculated and the calibration curve was drawn after internal verification (1000 bootstrap resampling) to evaluate the consistency of the nomogram prediction model [18]. The nomogram was then externally verified using the data of 198 HBV-ACLF patients collected from April 2020 to December 2021. In all comparisons, the results were considered statistically significant at *p* < 0.05.

## Results

### Demographics and characteristics of the enrolled patients

Study patients were divided into the survival (111 patients) and death/non-survival group (70 patients), based on survival condition at 90 days. The majority of patients in both groups were men (85.6% vs. 80.0%, respectively). The patients in the non-survival group were older than those in the survival group (52.84 ± 11.63 vs. 45.38 ± 11.25 years, *p* < 0.001), and non-survivors had a higher incidence of complications such as infection, UGB, ascites, and HE compared to the survivors (*p* = 0.004, *p* = 0.003, *p* = 0.044, and *p* < 0.001, respectively). The demographic characteristics of the participants are shown in Table 1.

**Table 1:** Patient demographics and characteristics

Variables	Survival (*n* = 111)	Non-survival (*n* = 70)	*p*
Sex (male)	95	56	0.325
Age (years)	45.38 ± 11.25	52.84 ± 11.63	< 0.001^*#^
BMI (kg/m^2^)	24.20 ± 3.11	23.26 ± 3.42	0.059^#^
HE	5	32	< 0.001^*^
Ascites	83	61	0.044^*^
UGB	3	10	0.003^*^
Infection	32	35	0.004^*^
WBC (10^9^/L)	6.70 (4.90–8.90)	6.80 (5.10–9.20)	0.933
Hb (g/L)	126.55 ± 18.84	125.40 ± 20.62	0.700^#^
PLT (10^9^/L)	111.00 (77.00–149.00)	103.00 (66.75–137.50)	0.186
ALT (U/L)	322.00 (131.00–673.00)	323.00 (108.25–549.25)	0.795
AST (U/L)	180.00 (93.00–362.00)	211.50 (106.00–407.75)	0.290
Albumin (g/L)	31.24 ± 4.40	31.10 ± 3.38	0.815^#a^
TBIL (µmol/L)	322.30 (264.40–393.90)	367.30 (295.38–451.60)	0.007^*^
Cr (µmol/L)	62.00 (55.00–72.00)	59.50 (52.00–73.50)	0.372
TC (mmol/L)	2.13 (1.82–2.62)	2.10 (1.62–2.76)	0.441
TG (mmol/L)	1.36 (1.11–1.75)	1.18 (0.98–1.63)	0.029^*^
HDL-C (mmol/L)	0.17 (0.10–0.23)	0.20 (0.14–0.27)	**0.017**
LDL-C (mmol/L)	0.64 (0.28–1.15)	0.91 (0.48–1.31)	0.070
AFP (ng/mL)	154.00 (49.10–365.80)	56.00 (18.08–176.30)	< 0.001^*^
INR	1.85 (1.68–2.09)	2.26 (1.90–2.91)	< 0.001^*^
D-dimer (ug/L)	1385.00 (766.00–2854.00)	2890.50 (1932.50–4045.25)	< 0.001^*^
Sodium (mmol/L)	137.00 (135.00–139.00)	137.00 (134.00–140.00)	0.432
Log_10_HBV-DNA (IU/ml)	5.17 ± 1.71	5.41 ± 2.20	0.432^#a^

*Abbreviations*: *BMI* body mass index, *HE* hepatic encephalopathy, *UGB* upper gastrointestinal bleeding, *WBC* white blood cell, *Hb* hemoglobin, *PLT* platelet, *ALT* alanine aminotransferase, *AST* aspartate aminotransferase, *TBIL* total bilirubin, *Cr* creatinine, *TC* total cholesterol, *TG* triglyceride, *HDL-C* high-density lipoprotein cholesterol, *LDL-C* low-density lipoprotein cholesterol, *AFP* alpha fetoprotein, *INR* international normalized ratio

^*^p value < 0.05; ^#^conforms to normal distribution on the SK test of normality; ^a^indicates that Levene’s test for equality of variances are not uniform, two independent T' tests of correction were applied

### Risk factors associated with 90-day mortality

Univariate analysis revealed that age, the occurrence of complications (i.e. infection, UGB, ascites, and HE), and the levels of TBIL, AFP, INR, TC, HDL-C, and D-dimer were significantly associated with 90-day mortality (*p* < 0.05). Results showed that there were no significant between-group differences in terms of sex, BMI, WBC, Hb, PLT, ALT, AST, albumin, Cr, TC, LDL-C, sodium, and Log_10_HBV-DNA (Table 1). Additionally, eleven risk factors identified on univariate analysis were screened using multivariate logistic regression analysis, which revealed that age (odds ratio [OR] 1.052, 95% confidence interval [CI] 1.012–1.095), HE (OR 9.059, 95% CI 2.604–31.514), TBIL (OR 1.006, 95% CI 1.002–1.011), INR (OR 1.014, 95% CI 1.004–1.023), and HDL-C (OR 1.080, 95% CI 1.017–1.146) were independent risk factors for 90-day mortality in patients with HBV-ACLF (Table 2).

**Table 2:** Multivariate logistic regression of risk factors of 90-day mortality

Variables	B value	SE	Wald	OR (95%CI)	*p*
Age	0.051	0.020	6.440	1.052 (1.012,1.095)	0.011*
HE	2.204	0.636	12.003	9.059 (2.604,31.514)	0.001*
Ascites	0.709	0.640	1.23	2.033 (0.580,7.120)	0.267
UGB	1.327	0.893	2.207	3.770 (0.655,21.705)	0.137
Infection	0.431	0.448	0.928	1.539 (0.640,3.700)	0.335
TBIL	0.006	0.002	7.273	1.006 (1.002,1.011)	0.007*
TG	0.659	0.379	3.021	1.933 (0.919,4.064)	0.082
HDL-C	0.077	0.030	6.397	1.08 (1.017,1.146)	0.011*
AFP	-0.002	0.001	0.168	0.998 (0.996,1.001)	0.168
INR	0.014	0.005	0.006	1.014 (1.004,1.023)	0.006*
D-dimer	0	0	0.33	1	0.566

*Abbreviations*: *HE* hepatic encephalopathy, *UGB* upper gastrointestinal bleeding, *TBIL* total bilirubin, *TG* triglyceride, *HDL-C* high-density lipoprotein cholesterol, *AFP* alpha fetoprotein, *INR* international normalized ratio

^*^p value < 0.05; Since the physiological changes of INR and HDL are small, in order to facilitate the observation of the fluctuations of small amplitude values, we magnified them by 100 times and put them into binary logistic analysis

### HBV-ACLF prognostic nomogram establishment and evaluation

**Fig. 2:**
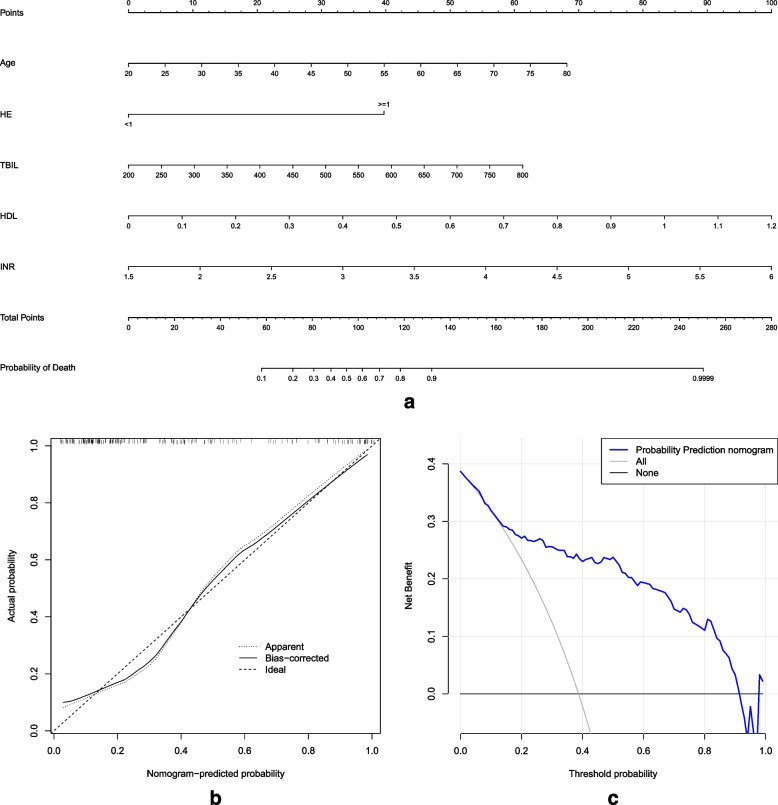
**a** The nomogram to predict 90-day mortality in patients with HBV-ACLF. Abbreviations: HBV-ACLF, hepatitis B virus-related acute-on-chronic liver failure; **b** Calibration curve of the prognostic nomogram in patients with HBV-ACLF. Notes: The x-axis represents the nomogram-based predicted mortality, and the y-axis represents the actual mortality. The diagonal dotted line represents an ideal model with no disparities between predicted mortality and the actual clinical result. The solid line represents the performance of the nomogram; **c** Decision curve analysis of the prognostic nomogram. Notes: The x-axis represents the threshold probability, and the y-axis represents the net benefit. The blue, gray, and black lines represent the prognostic nomogram, the assumption that all the patients will die, and the assumption that no patients will die, respectively. The decision curve analysis shows that the nomogram has a good overall net benefit in a wide range of threshold probabilities

The five abovementioned independent risk factors identified on multivariate analysis were introduced into the R software to establish a prognostic nomogram model for the 90-day mortality of patients with HBV-ACLF (Fig. 2a). The higher the score calculated from the sum of the specified scores of each predictor in the nomogram, the higher the probability of death.

The calibration curve of the nomogram for the prediction of 90-day mortality in patients with HBV-ACLF demonstrated a high level of agreement (Fig. 2b). The C-index for the prognostic nomogram was 0.866, and was confirmed to be 0.854 via bootstrapping verification, indicating a strong congruence between the findings on the nomogram and the actual results in the internal verification.

### Clinical usage

As shown in Fig. 2c, we performed decision curve analysis on the nomogram to estimate the net benefits of our nomogram to patients. The findings showed a significant net benefit for almost all threshold probabilities, especially the threshold probability between 2–91%.

### External validation of the prognostic nomogram

**Fig. 3:**
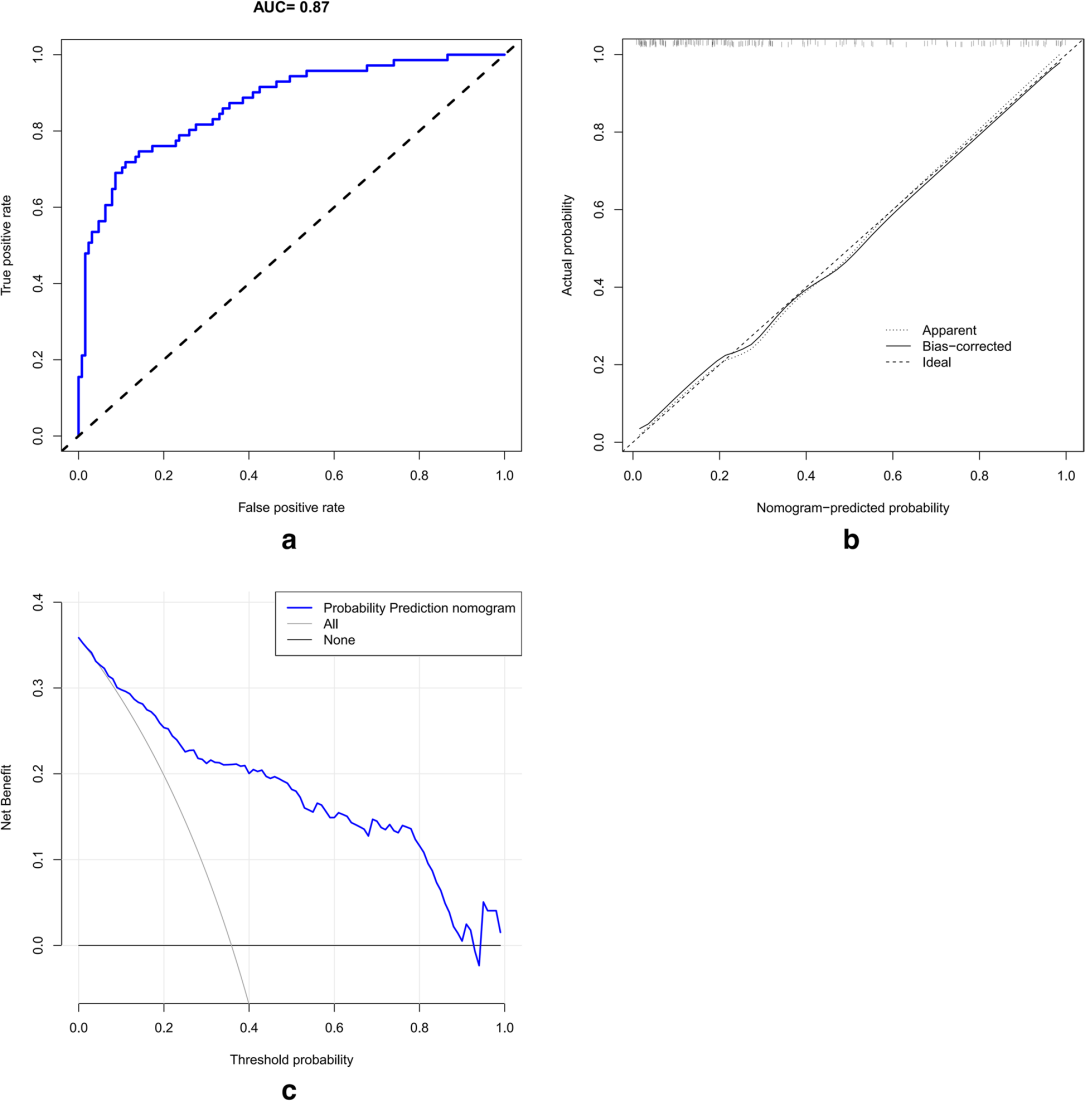
The ROC curve (**a**), calibration curve (**b**), and decision curve (**c**) of the prognostic nomogram in the external validation cohort

In the external set, the prognostic nomogram also showed good discrimination (AUC = 0.870) (Fig. 3a). As shown in Fig. 3b, the calibration curve was close to the ideal curve, indicating strong congruence between the predicted probability and the actual probability in the external set. Additionally, the decision curve analysis (Fig. 3c) suggested that the prognostic nomogram also provided net benefit in almost all of the threshold probability range.

**Fig. 4:**
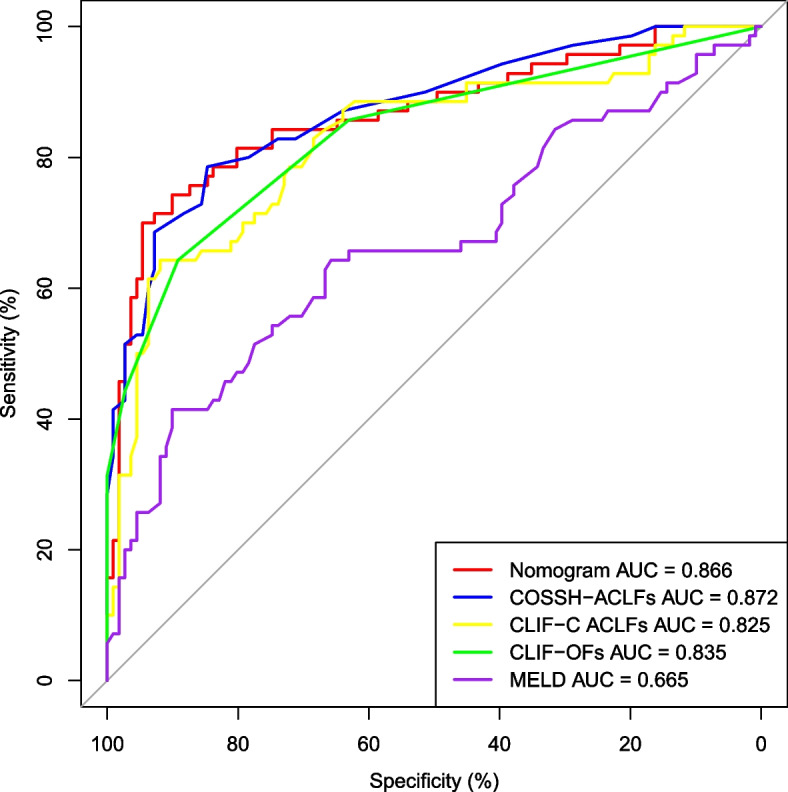
Comparison of abilities between the nomogram and other scoring systems in predicting the 90-day mortality of patients with HBV-ACLF

We plotted the ROCs to analyze and compare the predictive values of the nomogram, and COSSH-ACLFs in estimating the 90-day mortality of patients with HBV-ACLF (Fig. 4). The predictive ability of the nomogram was approximately comparable to that of the COSSH-ACLFs (AUC 0.866 vs. 0.872, *p* = 0.099).

## Discussion

ACLF is characterized by a rapid progression of multiple organ failure and a low survival rate, making it an urgent global health problem [4, 19]. In Asia, HBV infection and activation are the main causes of ACLF [20]. Further, the mortality of patients diagnosed with HBV-ACLF at admission is high, necessitating further research. It is crucial to develop an accurate and feasible mortality risk prediction model to enable the application of relevant interventions. This will aid in guiding the clinical decision making of medical staff, as well as in ameliorating patient outcomes.

Nowadays, nomograms are widely used as prognostic tools in medical studies. Due to their high accuracy, they play important roles in clinical decision making. In this single-center study, we screened five independent risk factors (age, TBIL, INR, HE, and HDL-C) and established a nomogram to predict 90-day mortality in patients with HBV-ACLF.

First, both the CLIF-C ACLFs and COSSH-ACLFs already include age in their prognostic scoring systems, and both consider old age as a risk factor for poor prognosis in patients with HBV-ACLF. This is logical, as an increase in age leads to significant decreases in liver volume, blood flow, and enzyme metabolism, which reduce the liver’s tolerance to diseases [21]. Second, high serum levels of INR and TBIL generally indicate coagulation failure and liver failure, respectively. Many prognostic studies have reported that the abovementioned parameters are indicators of survival in patients with liver diseases [22, 23]. Furthermore, the parameters are widely used in prognostic scores for patients with ACLF [14, 24, 25]. Additionally, patients with HE, especially those with ACLF, reportedly have higher mortality rates [1, 26]. Verma et al. previously revealed that the presence of HE was independently associated with mortality in patients with ACLF, and that patient mortality tends to increase with higher HE grades [27].

Importantly, HDL-C level, which is not included in any of the currently used prediction models, was considered a risk factor in our prognostic nomogram. It is well-known that a low HDL-C level is an independent predictor of cardiovascular risk [28, 29], and some recent studies have shown that blood lipid levels are decreased in patients with inflammatory diseases [20]. The possible underlying mechanism involves the binding of HDL-C particles to lipopolysaccharides, resulting in the inhibition of the activation of inflammatory factors [30]. According to a cross-sectional study, low HDL-C level is a poor prognostic factor for chronic liver failure [31]. In our study, HDL-C levels in patients with HBV-ACLF were lower than the established normal values (0.78–1.81 mmol/L) in both the survival (0.17, 95% CI 0.10–0.23) and non-survival groups (0.20, 95% CI 0.14–0.27). However, in our prognostic nomogram, we concluded that a lower HDL-C level was beneficial for the survival of patients with HBV-ACLF. This result is contrary to the conventional view that HDL-C is a “good cholesterol” [32, 33]. Indeed, recent studies have reported that HDL-C may not always be a “good cholesterol.” Silbernagel et al. found that the protective effect of HDL-C disappeared in patients with unstable coronary heart disease [34]. Moreover, some studies have shown that, unlike in healthy individuals, HDL-C does not exert any vascular protective effects in patients with kidney disease or diabetes; in fact, increased levels of HDL-C may even have harmful effects [35–37]. We speculated that in a diseased state, HDL-C may have a negative impact on the human body, owing to disease-induced dysfunction. Therefore, in patients with HBV-ACLF, higher HDL-C levels may be more detrimental to patient survival. Nevertheless, more intensive basic experiments and mechanistic research are needed to clarify the abovementioned speculation. It is still unknown whether HDL-C plays a role in the course of HBV-ACLF.

As observed, the areas under the ROCs of the nomogram and COSSH-ACLFs were very similar. Hence, it could be considered that the predictive abilities of the nomogram and COSSH-ACLFs are comparable. The COSSH-ACLFs is a complex score based on organ failure, comprising 11 parameters which are complicated to evaluate [15]. As Chen et al. stated in their article, pulse oxygen saturation or arterial blood gas analysis were recently used in their center to routinely evaluate the respiratory function of patients with ACLF [38]. The complexity of the COSSH-ACLFs significantly limits its application. In the present study, five independent risk factors were selected to build a prognostic nomogram that is simple, intuitive, precise, and universal.

However, the present study has several limitations. First, this was a single-center, retrospective study, which lead to inevitable selection bias, and the majority of the participants were men. Moreover, we excluded patients who were lost follow-up or with incomplete data, which may have affected the efficacy of the nomogram. Second, the course of ACLF changes dynamically. The data in this study were collected at the time of diagnosis rather than at consecutive time points. Moreover, subsequent changes in treatment and nursing care may lead to variations in relevant parameters. Third, further studies are required to confirm whether HDL-C can be used as a prognostic predictor in patients with HBV-ACLF, and to clarify the mechanism of HDL-C in the pathogenesis of HBV-ACLF. Lastly, although the robustness of our prognostic nomogram was checked via internal validation (bootstrap testing) and external validation, external evaluation with a larger, multi-center population is still required.

## Conclusion

In this study, we established and validated a simple, prognostic nomogram to predict 90-day mortality in patients with HBV-ACLF. Additionally, this nomogram can be used for early detection of patients with HBV-ACLF, making clinical decisions, and guiding the allocation of medical resources. Although our nomogram was internally validated by internal verification (1000 bootstrap resampling) and assessed by using another validation cohort, this nomogram still requires external validation before its use can be popularized.

## Data Availability

The data supporting the findings of this study are available from The First Affiliated Hospital, Zhejiang University School of Medicine; however, restrictions apply to the availability of these data, which were used under license for the current study, and so are not publicly available. Data are however available from the authors upon reasonable request and with permission of The First Affiliated Hospital, Zhejiang University School of Medicine.

## References

[1] Khanam A, Kottilil S (2021). Acute-on-chronic liver failure: pathophysiological mechanisms and management. Front Med (Lausanne).

[2] Sarin SK (2019). Acute-on-chronic liver failure: consensus recommendations of the Asian Pacific association for the study of the liver (APASL): an update. Hepatol Int.

[3] Yuen MF (2018). Hepatitis B virus infection. Nat Rev Dis Primers.

[4] Tang LSY (2018). Chronic hepatitis B infection: a review. JAMA.

[5] Chen EQ (2015). Early warning and clinical outcome prediction of acute-on-chronic hepatitis B liver failure. World J Gastroenterol.

[6] Wu D (2018). HINT: a novel prognostic model for patients with hepatitis B virus-related acute-on-chronic liver failure. Aliment Pharmacol Ther.

[7] Liu J, Fan D (2007). Hepatitis B in China. Lancet.

[8] Zhao RH (2018). Acute-on-chronic liver failure in chronic hepatitis B: an update. Expert Rev Gastroenterol Hepatol.

[9] Huang K (2019). Artificial liver support system therapy in acute-on-chronic hepatitis B liver failure: classification and regression tree analysis. Sci Rep.

[10] Kribben A (2012). Effects of fractionated plasma separation and adsorption on survival in patients with acute-on-chronic liver failure. Gastroenterology.

[11] Volarevic V (2018). Ethical and safety issues of stem cell-based therapy. Int J Med Sci.

[12] Goosmann L (2021). Liver transplantation for acute-on-chronic liver failure predicts post-transplant mortality and impaired long-term quality of life. Liver Int.

[13] Wiesner R (2003). Model for end-stage liver disease (MELD) and allocation of donor livers. Gastroenterology.

[14] Jalan R (2014). Development and validation of a prognostic score to predict mortality in patients with acute-on-chronic liver failure. J Hepatol.

[15] Wu T (2018). Development of diagnostic criteria and a prognostic score for hepatitis B virus-related acute-on-chronic liver failure. Gut.

[16] Kamath PS, Kim WR (2007). The model for end-stage liver disease (MELD). Hepatology.

[17] Vickers AJ, Elkin EB (2006). Decision curve analysis: a novel method for evaluating prediction models. Med Decis Making.

[18] Pencina MJ, D'Agostino RB (2004). Overall C as a measure of discrimination in survival analysis: model specific population value and confidence interval estimation. Stat Med.

[19] Shah S, Goldberg DS (2021). Acute-on-chronic liver failure: update on pathogenesis, therapeutic targets, predictive models, and liver transplantation. Curr Opin Gastroenterol.

[20] Mecatti GC, Messias MCF, de Oliveira Carvalho P (2020). Lipidomic profile and candidate biomarkers in septic patients. Lipids Health Dis.

[21] Tajiri K, Shimizu Y (2013). Liver physiology and liver diseases in the elderly. World J Gastroenterol.

[22] Cai JJ (2019). Characteristics, risk factors, and adverse outcomes of hyperkalemia in acute-on-chronic liver failure patients. Biomed Res Int.

[23] Peng Y (2021). Prediction and risk factors for prognosis of cirrhotic patients with hepatic encephalopathy. Gastroenterol Res Pract.

[24] Choudhury A (2017). Liver failure determines the outcome in patients of acute-on-chronic liver failure (ACLF): comparison of APASL ACLF research consortium (AARC) and CLIF-SOFA models. Hepatol Int.

[25] Said A (2004). Model for end stage liver disease score predicts mortality across a broad spectrum of liver disease. J Hepatol.

[26] Cordoba J (2014). Characteristics, risk factors, and mortality of cirrhotic patients hospitalized for hepatic encephalopathy with and without acute-on-chronic liver failure (ACLF). J Hepatol.

[27] Verma N (2021). Dynamic assessments of hepatic encephalopathy and ammonia levels predict mortality in acute-on-chronic liver failure. Hepatol Int.

[28] Cziraky MJ, Watson KE, Talbert RL (2008). Targeting low HDL-cholesterol to decrease residual cardiovascular risk in the managed care setting. J Manag Care Pharm.

[29] Lu Q (2013). Low HDL-C predicts risk and PCI outcomes in the Han Chinese population. Atherosclerosis.

[30] Levine DM (1993). In vivo protection against endotoxin by plasma high density lipoprotein. Proc Natl Acad Sci U S A.

[31] Trieb M (2020). HDL-related biomarkers are robust predictors of survival in patients with chronic liver failure. J Hepatol.

[32] Gong X (2022). Associations of lipid profiles with the risk of ischemic and hemorrhagic stroke: a systematic review and meta-analysis of prospective cohort studies. Front Cardiovasc Med.

[33] Sirtori CR (2019). HDL therapy today: from atherosclerosis, to stent compatibility to heart failure. Ann Med.

[34] Silbernagel G (2013). High-density lipoprotein cholesterol, coronary artery disease, and cardiovascular mortality. Eur Heart J.

[35] Sorrentino SA (2010). Endothelial-vasoprotective effects of high-density lipoprotein are impaired in patients with type 2 diabetes mellitus but are improved after extended-release niacin therapy. Circulation.

[36] März W (2017). HDL cholesterol: reappraisal of its clinical relevance. Clin Res Cardiol.

[37] Speer T (2013). Abnormal high-density lipoprotein induces endothelial dysfunction via activation of Toll-like receptor-2. Immunity.

[38] Chen JF (2021). Derivation and validation of a nomogram for predicting 90-day survival in patients with HBV-Related Acute-on-Chronic Liver Failure. Front Med (Lausanne).

